# Astragaloside IV and Ferroptosis Modulation in Pathological Cardiac Hypertrophy: Current Evidence, an Integrative Hypothesis, and Future Directions

**DOI:** 10.1002/prp2.70334

**Published:** 2026-10-16

**Authors:** Pengbo Liu, Yongzhi Deng

**Affiliations:** ^1^ Department of Cardiovascular Surgery The Affiliated Cardiovascular Hospital of Shanxi Medical University, Shanxi Cardiovascular Hospital (Institute), Shanxi Clinical Medical Research Center for Cardiovascular Disease Taiyuan Shanxi China

**Keywords:** astragaloside IV, cardiac remodeling, cardiomyocyte ferroptosis, ferroptosis, Nrf2‐SLC7A11‐GPX4 signaling axis, pathological cardiac hypertrophy

## Abstract

Pathological cardiac hypertrophy is initially an adaptive response to sustained mechanical, neurohormonal, or metabolic stress; however, it can progress to fibrosis, contractile dysfunction, arrhythmia, and heart failure. Ferroptosis is a type of regulated cell death caused by iron‐dependent phospholipid peroxidation. Evidence consistent with ferroptosis, such as disrupted iron homeostasis, lipid peroxidation, mitochondrial dysfunction and impaired antioxidant defense, has been reported in experimental models of pathological cardiac hypertrophy; however, these findings do not by themselves establish ferroptosis as a cause of hypertrophic remodeling. Astragaloside IV (AS‐IV) is a cycloartane‐type triterpene saponin from Astragalus species that has shown anti‐hypertrophic, antioxidant, anti‐inflammatory, metabolic‐regulatory and mitochondrial‐protective effects in experimental cardiovascular models. AS‐IV has also shown anti‐ferroptotic effects in several cardiac and extra‐cardiac disease models by means of Nrf2‐centred antioxidant defense, regulation of SLC7A11/GPX4, p53 signaling, CD36‐dependent lipid handling, and 14‐3‐3η‐linked regulation of GPX4 stability. Nevertheless, whether ferroptosis inhibition directly contributes to the anti‐hypertrophic effect of AS‐IV in pathological cardiac hypertrophy remains unproven because direct causal validation in dedicated hypertrophy models is lacking. This review collects evidence on the biology of ferroptosis in pathological cardiac hypertrophy, the established anti‐hypertrophic pharmacology of AS‐IV, its reported anti‐ferroptotic effects, and relevant pharmacokinetic considerations. Based on this, we propose that AS‐IV may reduce the susceptibility of cardiomyocytes to ferroptosis and thus partially inhibit pathological cardiac remodeling; however, this is not yet a proven mechanism. Direct verification requires etiologically distinct hypertrophy models, multi‐modal ferroptosis measurements, targeted genetic or pharmacological disruption, dose–response and pharmacokinetic experiments, and cell‐type‐resolved analysis.

## 1 Introduction

When cardiomyocytes are exposed to prolonged mechanical or biochemical stress caused by hypertension, prolonged activation of neurohormones, or metabolic disorders, they enlarge through a process known as hypertrophy. Although this initial growth response helps maintain cardiac output under stress, prolonged pathological hypertrophy can lead to reduced contractility, myocardial fibrosis, and heart failure [1, 2, 3]. Although neurohormonal blockade has shown some clinical benefits, many patients still experience progression of hypertrophy‐related heart failure. Therefore, research has focused on identifying the molecular mechanisms that promote the transition from initial compensatory growth to maladaptive cardiac remodeling and developing targeted therapies.

Ferroptosis is an iron‐dependent form of regulated cell death characterized by excessive phospholipid peroxidation and distinct from apoptosis and other canonical cell‐death programs [4, 5, 6, 7, 8, 9]. Since its introduction in 2012, ferroptosis has been linked to various types of cardiovascular damage, such as myocardial infarction, ischemia–reperfusion injury, diabetic cardiomyopathy and heart failure [6, 10, 11, 12, 13, 14]. Evidence from iron‐overload models and conventional hypertrophy models further shows that ferroptosis‐associated changes can occur in pathological cardiac hypertrophy [11, 12, 15, 16, 17, 18, 19, 20, 21, 22, 23]. Several interventions that reduce experimental hypertrophy also decrease ferroptosis‐related injury, suggesting a possible mechanism; however, these observations alone do not prove that ferroptosis is the main cause of hypertrophic growth or that its inhibition is necessary for the anti‐hypertrophic effect [15, 16, 17, 18, 24].

Astragaloside IV (AS‐IV, also known as astragaloside A) is a cycloartane‐type triterpene saponin and one of the main active components of Astragalus membranaceus and other Astragalus species used in traditional Chinese medicine [25]. Experimental studies have shown that AS‐IV inhibits pathological cardiac hypertrophy in pressure‐overload, inflammatory, β‐adrenergic, hypoxic, developmental and diabetic models by means of calcium‐calcineurin signaling, NF‐κB/PGC‐1α, calpain‐1/mTOR, PKCβII/Egr‐1, calpain‐1/CaMKII, and EGR1‐SIRT1‐PPARα‐SCAD pathways [26, 27, 28, 29, 30, 31, 32]. Separately, AS‐IV has been reported to suppress ferroptosis‐related injury in cardiac models of anthracycline cardiotoxicity, diabetic cardiomyopathy, chronic heart failure, and ischemia–reperfusion injury, with additional mechanistic support from extra‐cardiac tissues [33, 34, 35, 36, 37, 38, 39, 40, 41, 42, 43, 44, 45, 46]. The two bodies of evidence provide a foundation for investigating whether ferroptosis regulation contributes to the anti‐hypertrophic effect of AS‐IV.

Notably, the evidence that AS‐IV attenuates pathological cardiac hypertrophy and the evidence that AS‐IV suppresses ferroptosis have largely developed in parallel. Direct experimental evidence showing that ferroptosis inhibition mediates the anti‐hypertrophic effect of AS‐IV in pathological cardiac hypertrophy models is currently unavailable. To our knowledge, no previous review has systematically integrated the three areas of evidence: ferroptosis in pathological cardiac hypertrophy, the established anti‐hypertrophic pharmacology of AS‐IV, and the anti‐ferroptotic effects of AS‐IV. Therefore, the purpose of this review is to (i) summarize the molecular mechanisms and regulation of ferroptosis; (ii) critically evaluate the evidence linking ferroptosis to pathological cardiac hypertrophy; (iii) review the experimentally established anti‐hypertrophic effects of AS‐IV; (iv) synthesize cardiac and extra‐cardiac evidence for AS‐IV‐mediated ferroptosis regulation; and (v) propose a testable mechanistic hypothesis and experimental system. Throughout the review, established findings are distinguished from inferred links and unconfirmed hypotheses.

## 2 Ferroptosis: Molecular Mechanisms and Regulatory Pathways

### 2.1 Iron Metabolism and the Labile Iron Pool

Ferroptosis requires intracellular bioavailable iron and is characterized by iron‐dependent phospholipid peroxidation [4, 5, 6]. Transferrin‐bound iron enters cells through transferrin receptor 1 (TFR1)‐mediated endocytosis. Intracellular iron can be stored in ferritin, exported by ferroportin, or retained in the labile Fe^2+^ pool, where it can promote Fenton chemistry and oxidative damage [5, 6, 47]. Ferroptotic lipid peroxidation involves oxidation of polyunsaturated phospholipids, with ACSL4 and LPCAT3 contributing to the generation of oxidation‐prone phospholipid substrates [5, 6, 7, 8, 9]. NCOA4‐mediated ferritinophagy can release ferritin‐stored iron into the labile iron pool and thereby increase ferroptosis susceptibility [6]. Cardiac function is sensitive to disturbances in iron homeostasis, and both iron deficiency and iron overload can be detrimental [6, 12]. In the heart, disturbances in mitochondrial function, iron homeostasis, lipid redox balance, and antioxidant defense can have important functional consequences. Experimental cardiac disease models have documented ferroptosis‐associated injury under conditions of iron dysregulation and oxidative stress [6, 11, 12]. These observations support the relevance of ferroptosis to cardiac injury but do not establish that the myocardium is intrinsically more susceptible to ferroptosis than other tissues.

### 2.2 The SLC7A11–Glutathione–GPX4 Defense Axis and Complementary Ferroptosis Defense Pathways

The system xc^−^–GSH–GPX4 axis is a central ferroptosis‐defense pathway [7, 8, 9]. SLC7A11/SLC3A2‐mediated cystine uptake provides the necessary GSH for GPX4 to reduce phospholipid hydroperoxides and prevent ferroptosis of the membrane [5, 6]. Erastin inhibits the system xc^−^ and RSL3 directly inhibits GPX4; both are commonly used experimental ferroptosis inducers. Ferrostatin‐1 and liproxstatin‐1 inhibit ferroptotic lipid peroxidation and are frequently employed as ferroptosis inhibitors [4, 5, 6]. Other GPX4‐independent ferroptosis‐defense systems include the FSP1‐CoQ10 axis and the GCH1‐BH4 pathway [6]. GPX4‐dependent detoxification of phospholipid hydroperoxides is a central defense against ferroptosis [5, 6]. Ferroptosis susceptibility therefore reflects the balance among bioavailable iron, oxidizable phospholipids, and cellular antioxidant defenses.

### 2.3 Transcriptional and Stress‐Responsive Control of Ferroptosis

Stress‐responsive transcriptional programs regulate cellular susceptibility to ferroptosis. Nrf2 coordinates antioxidant and iron‐handling programs and can modulate ferroptosis susceptibility through regulation of genes involved in redox defense and iron metabolism [5, 6]. The role of p53 in ferroptosis is context dependent. One established pro‐ferroptotic mechanism involves repression of SLC7A11, thereby limiting cystine uptake and weakening glutathione‐dependent antioxidant defense [5, 6]. BACH1 can increase ferroptosis susceptibility by repressing genes involved in glutathione synthesis and iron storage/export, including Gclm, Slc7a11, Fth1, Ftl1, and Slc40a1. In experimental models, Bach1 deficiency was associated with reduced ferroptosis susceptibility and protection against myocardial ischemic injury [10].

### 2.4 Cardiac Relevance of Ferroptosis

Cardiomyocytes have high energy demands and are easily damaged by disorders of mitochondrial function, oxidative balance and iron homeostasis. Experimental cardiac disease models have linked iron dysregulation, lipid peroxidation, and impaired antioxidant defense with ferroptosis‐associated myocardial injury [6, 11, 12]. However, the above observations do not prove that the myocardium is intrinsically more susceptible to ferroptosis than other tissues, and the loss of ferroptotic cardiomyocytes does not necessarily lead to cardiac decompensation. Therefore, cardiac ferroptosis should be assessed by model‐specific biochemical, morphological and rescue experiments rather than based solely on tissue characteristics. Recent cardiac studies have further extended this evidence base to mechanistically defined myocardial ischemia–reperfusion injury and to human diabetic heart failure tissue, while emphasizing that ferroptosis‐related changes remain disease‐ and context‐dependent [48, 49]. A recent international review of ferroptosis in heart failure similarly emphasizes convergent abnormalities in iron handling, antioxidant defense, phospholipid peroxidation, and mitochondrial stress across multiple heart‐failure models [50]. The essential molecular machinery and primary regulatory pathways of ferroptosis are shown in Figure 1.

**FIGURE 1:**
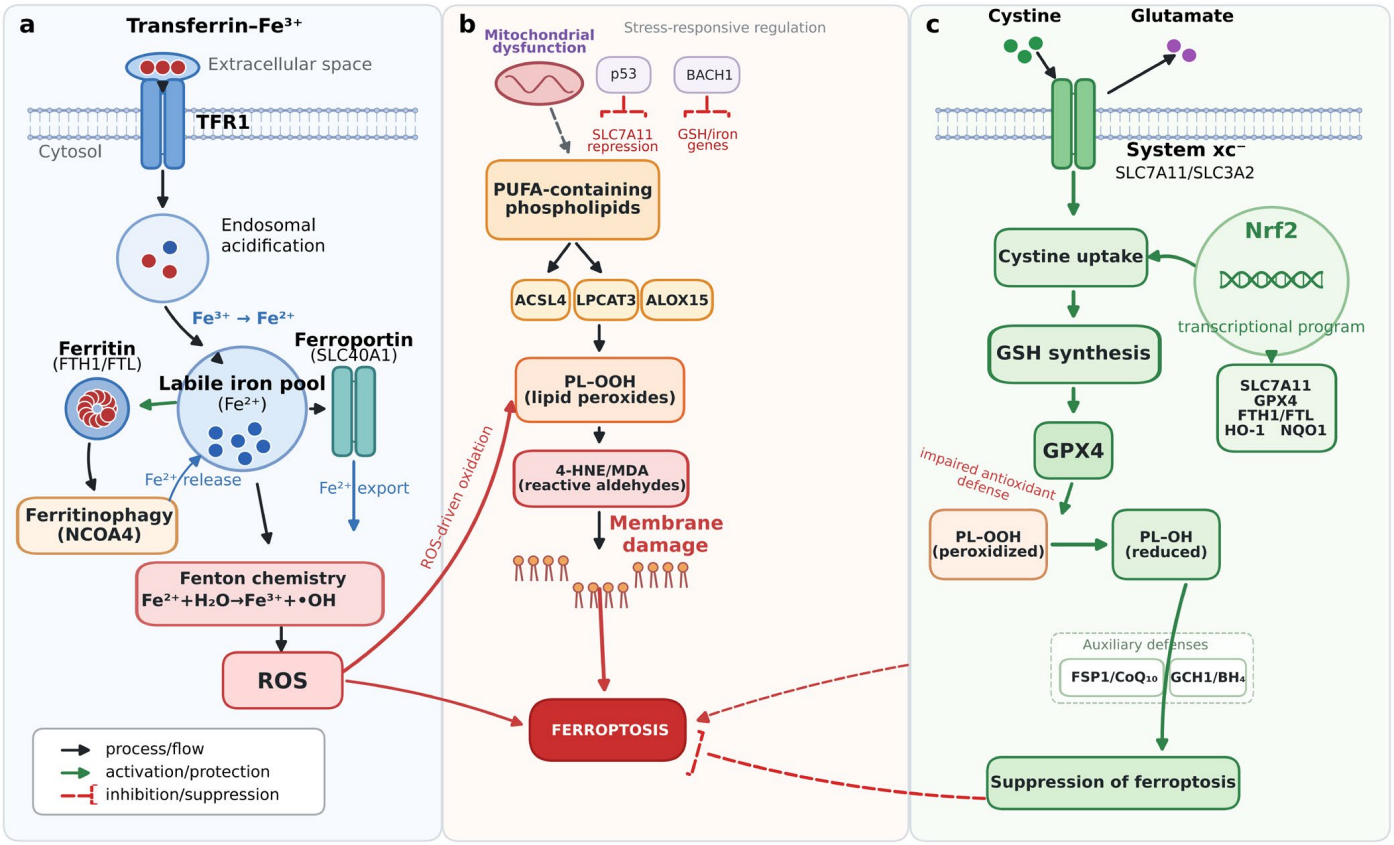
Molecular machinery of ferroptosis. (a) Iron uptake, ferritin turnover, ferroportin‐mediated export, and NCOA4‐dependent ferritinophagy regulate the labile Fe^2+^ pool and promote Fenton chemistry and ROS generation. (b) ACSL4‐ and LPCAT3‐dependent phospholipid remodeling generates PUFA‐containing phospholipids that undergo enzymatic and non‐enzymatic lipid peroxidation, producing phospholipid hydroperoxides and reactive aldehydes that damage cellular membranes. Stress‐responsive regulators, including p53 and BACH1, influence iron, lipid, and antioxidant balance. (c) Ferroptosis‐defense mechanisms include the system xc^−^–GSH–GPX4 axis, FSP1/CoQ10, GCH1/BH4, ferritin/ferroportin‐dependent iron handling, and the Nrf2 transcriptional program. Arrows indicate activation, protection, or process flow; blunt‐ended lines indicate inhibition or suppression; dashed lines indicate indirect or context‐dependent regulation. 4‐HNE, 4‐hydroxynonenal; ACSL4, acyl‐CoA synthetase long‐chain family member 4; ALOX15, arachidonate 15‐lipoxygenase; BACH1, BTB and CNC homology 1; BH4, tetrahydrobiopterin; CoQ10, coenzyme Q10; FSP1, ferroptosis suppressor protein 1; FTH1, ferritin heavy chain 1; FTL, ferritin light chain; GCH1, GTP cyclohydrolase 1; GPX4, glutathione peroxidase 4; GSH, glutathione; HO‐1, heme oxygenase 1; LPCAT3, lysophosphatidylcholine acyltransferase 3; MDA, malondialdehyde; NCOA4, nuclear receptor coactivator 4; NQO1, NAD(P)H quinone dehydrogenase 1; Nrf2, nuclear factor erythroid 2‐related factor 2; PL‐OH, reduced phospholipid; PL‐OOH, phospholipid hydroperoxide; PUFA, polyunsaturated fatty acid; ROS, reactive oxygen species; SLC3A2, solute carrier family 3 member 2; SLC40A1, solute carrier family 40 member 1; SLC7A11, solute carrier family 7 member 11; system xc^−^, cystine/glutamate antiporter; TFR1, transferrin receptor 1.

## 3 Ferroptosis Participation in Pathological Cardiac Hypertrophy

### 3.1 Direct Evidence From Iron Overload and Genetic Manipulation Studies

Cardiomyocyte‐specific Fth‐deletion mice were initially viable; however, after high‐iron challenge, they developed severe cardiac injury with hypertrophic cardiomyopathy, myocardial GSH depletion and increased lipid peroxidation. Ferrostatin‐1 attenuated the cardiac phenotype, and cardiomyocyte‐specific Slc7a11 overexpression restored GSH and enhanced resistance to ferroptosis‐associated injury [11]. The above results support a causal role for impaired ferritin/SLC7A11‐dependent ferroptosis defense in this genetic iron‐overload model, but they should not be applied to all types of pathological cardiac hypertrophy. More broadly, iron overload can cause cardiac injury, and ferroptosis‐related mechanisms have been implicated in experimental iron‐overload cardiomyopathy [6, 11].

### 3.2 Evidence From Neurohormonal Stimulation and Pressure‐Overload Disease Models

Alterations in ferroptosis have been observed in several experimental models of pathological cardiac hypertrophy. Paeonol reduced hypertrophy and ferroptosis‐related changes in Angiotensin II (AngII)‐stimulated cardiomyocytes [15]. Mocetinostat reduced hypertrophic remodeling and attenuated changes in lipid peroxidation, iron accumulation, GPX4, SLC7A11, FTH1, and HO‐1 in phenylephrine‐treated primary cardiomyocytes and transverse aortic constriction (TAC) mice [16]. Altered glycosylation of CD147 in TAC mice modified pathological remodeling via TRAF2‐TAK1 signaling and was associated with changes in oxidative injury and ferroptosis [17]. In TAC‐induced heart failure, N‐lactoyl‐phenylalanine reduced hypertrophy, fibrosis, apoptosis, and ferroptosis‐related injury, and improved MFN2‐related mitochondrial remodeling [18]. Pathological hypertrophy or heart‐failure‐related remodeling coexists with ferroptosis‐associated biochemical and molecular alterations in all these models. However, the extent to which ferroptosis is causally related to hypertrophic growth varies among models and has not been uniformly determined [15, 16, 17, 18].

More recent studies have expanded this model‐specific evidence base. PM2.5‐associated cardiac hypertrophy has been linked to disturbed iron homeostasis, selective‐autophagy crosstalk, and ferroptosis [19]. In pressure‐overload heart failure, ACSL4 upregulation and altered phospholipid metabolism were associated with ferroptosis, while cardiomyocyte‐specific Acsl4 deletion or pharmacological inhibition attenuated ferroptosis‐associated injury and adverse cardiac remodeling [20]. Additional AngII‐ and/or pressure‐overload studies have linked MARCHF6‐dependent ACSL4 degradation [21], PACS2/SIRT1‐associated regulation [22], and FBXO10‐mediated ACSL4 destabilization [23] to parallel changes in cardiomyocyte ferroptosis and hypertrophic remodeling. Additional mechanistic studies further support model‐dependent involvement of ferroptosis in pathological hypertrophic remodeling. In AngII‐induced cardiac hypertrophy, PKD knockdown reduced hypertrophy and ferroptosis‐associated injury in association with suppression of JNK/p53 signaling [51]. In a separate AngII‐induced remodeling model, genetic or molecular suppression of P2X7R attenuated myocardial ferroptosis and adverse remodeling, with HuR‐dependent regulation of GPX4 and HO‐1 mRNA stability implicated in the response [52]. More recently, the Irx3–Etfa transcriptional axis was shown to attenuate AngII‐ and TAC‐associated hypertrophic remodeling and ferroptosis; Etfa knockdown reduced the protective effects of Irx3, providing additional perturbation‐based evidence linking mitochondrial regulation to ferroptosis in pathological cardiac hypertrophy [53]. Collectively, these studies strengthen model‐specific evidence that ferroptosis‐related regulation can influence pathological cardiac remodeling, but they do not establish that ferroptosis inhibition mediates the anti‐hypertrophic effect of AS‐IV.

### 3.3 Shared Upstream Mechanisms Linking Hypertrophic Signaling and Ferroptosis

Calcium‐calcineurin and calpain‐related signaling are established contributors to pathological hypertrophic remodeling and are modulated by AS‐IV in several hypertrophy models [27, 29, 31]. Separately, mitochondrial dysfunction and oxidative stress accompany ferroptosis‐associated injury in cardiac disease models [12, 54, 55, 56]. Direct evidence that calcium‐calcineurin or calpain‐CaMKII signaling mechanistically drives ferroptosis during pathological cardiac hypertrophy remains limited.

Inflammatory signaling is an important component of pathological cardiac remodeling, and inflammatory changes often coexist with ferroptosis‐associated injury. However, the current evidence summarized here does not establish a specific TLR4/NF‐κB–ferroptosis causal axis in pathological cardiac hypertrophy.

Pathological cardiac hypertrophy is accompanied by metabolic remodeling, including altered fatty‐acid utilization [3]. AS‐IV has been reported to modulate NF‐κB/PGC‐1α signaling in isoproterenol‐induced hypertrophy [28] and EGR1‐SIRT1‐PPARα‐SCAD signaling in pressure‐overload hypertrophy [32]. Whether these metabolic changes directly alter ferroptosis susceptibility during pathological hypertrophy remains untested.

Oxidative stress, GSH depletion, lipid peroxidation, and impaired GPX4‐dependent defense are central features of ferroptosis and have been reported in several cardiac and hypertrophy‐related experimental models [6, 11, 12, 15, 16]. Their co‐occurrence with hypertrophic remodeling supports biological overlap but does not by itself establish ferroptosis as a causal mediator of hypertrophy.

### 3.4 Ferroptosis as a Therapeutic Target in Hypertrophic Heart Disease

Evidence of ferroptosis in pathological hypertrophy has drawn attention to therapeutic strategies based on natural compounds and drug repurposing. Paeonol and mocetinostat reduce ferroptosis‐related changes and hypertrophic remodeling simultaneously in experimental models [15, 16]. The LuQi formula activates Nrf2/GPX4 signaling and inhibits ferroptosis in heart failure cardiomyocytes [56]. Optimized Shengmai Powder ameliorates post‐infarction heart failure with myocardial hypertrophy and fibrosis by regulating the cGMP‐PKG‐GPX4 axis [55]. TMEM43 protects septic hearts from ferroptosis‐related injury and maintains GPX4/SLC7A11 defense [54]. Research on natural products that inhibit myocardial hypertrophy has also identified ferroptosis‐related regulation as a possible cardioprotective mechanism [24]. Collectively, these studies support ferroptosis as a candidate therapeutic target in cardiac remodeling [57]. However, none directly demonstrates that ferroptosis inhibition mediates the anti‐hypertrophic effect of AS‐IV in a dedicated pathological cardiac hypertrophy model. Accordingly, the AS‐IV–ferroptosis–hypertrophy connection should be regarded as a testable mechanistic hypothesis rather than an established therapeutic mechanism. The present evidence of ferroptosis in pathological cardiac hypertrophy is shown in Table 1.

**TABLE 1:** Evidence linking ferroptosis to pathological cardiac hypertrophy.

Model	Ferroptosis evidence	Intervention/causal tool	References
Cardiomyocyte‐specific ferritin H knockout + high‐iron diet (mice)	High‐iron challenge in cardiac Fth‐deficient mice causes GSH depletion, lipid peroxidation, cardiomyocyte ferroptosis, and hypertrophic cardiomyopathy	Fer‐1 rescues the phenotype; cardiomyocyte Slc7a11 overexpression restores ferroptosis defense	[11]
AngII‐induced cardiac hypertrophy (mice; primary cardiomyocytes)	Hypertrophy is accompanied by Fe^2+^ and lipid‐ROS accumulation, increased MDA/ROS, reduced GSH, and decreased xCT/GPX4	Paeonol attenuates both hypertrophy and ferroptosis‐associated changes; Fer‐1 suppresses AngII‐induced ferroptotic cell injury, but mediation of hypertrophy is not directly established	[15]
Phenylephrine‐treated primary cardiomyocytes; TAC (mice)	Lipid peroxidation and iron accumulation increase, with reduced GPX4, SLC7A11, FTH1, and HO‐1	Mocetinostat suppresses hypertrophy and ferroptosis while activating Nrf2; Nrf2 inhibition with ML385 attenuates these protective effects	[16]
TAC (mice)	Cardiac CD147 glycosylation enhances oxidative stress and ferroptosis‐associated changes alongside hypertrophy and fibrosis	Glycosylation‐defective CD147 attenuates remodeling and ferroptosis‐associated changes; CD147‐TRAF2 interaction activates TAK1 signaling	[17]
TAC‐induced heart failure (mice)	Hypertrophy, fibrosis, apoptosis, ferroptosis‐associated changes, and MFN2 downregulation coexist	N‐Lac‐Phe improves cardiac function and attenuates hypertrophy, fibrosis, apoptosis, and ferroptosis‐associated changes, accompanied by restoration of MFN2 and AMPK/PGC‐1α‐associated mitochondrial signaling	[18]
Post‐myocardial‐infarction heart failure (rats)	Myocardial hypertrophy, fibrosis, lipid peroxidation, and ferroptosis‐associated changes coexist with suppression of cGMP‐PKG‐GPX4 signaling	Optimized New Shengmai Powder attenuates remodeling and ferroptosis while enhancing cGMP‐PKG‐GPX4 signaling; direct mediation of hypertrophy by ferroptosis was not tested	[55]
TAC‐induced heart failure (mice; neonatal rat cardiomyocytes)	Ferroptosis‐associated iron/redox alterations and mitochondrial abnormalities accompany myocardial hypertrophy and fibrosis	LuQi formula suppresses ferroptosis‐associated changes and attenuates hypertrophy/fibrosis in association with Nrf2/GPX4 pathway activation	[56]
PM2.5‐associated cardiac hypertrophy	Disturbed iron homeostasis, lipid peroxidation, and ferroptosis‐associated changes accompany cardiac hypertrophy; selective‐autophagy crosstalk involving iron handling was implicated	NCOA4 knockdown attenuated PM2.5‐induced iron‐homeostasis disruption and ferroptosis‐associated changes, supporting involvement of NCOA4‐dependent ferritinophagy in this model	[19]
TAC‐induced pressure‐overload heart failure	ACSL4 and oxidizable phospholipid species are increased; ferroptosis‐associated lipid injury accompanies pressure‐overload remodeling	Cardiomyocyte‐specific Acsl4 deletion and pharmacological inhibition reduce ferroptosis‐associated injury and adverse remodeling; Acsl4 overexpression worsens the phenotype	[20]
AngII‐treated cardiomyocytes; TAC	Hypertrophy, oxidative stress, and ferroptosis‐associated changes occur together with altered PACS2/SIRT1–Nrf2/HO‐1 regulation	PACS2 silencing reduces hypertrophy and ferroptosis‐associated changes; SIRT1 inhibition partially reverses the protective phenotype	[22]
Phenylephrine‐treated cardiomyocytes; TAC	Reduced FBXO10 and increased ferroptosis susceptibility accompany hypertrophic stress; ACSL4 is identified as a downstream ferroptosis‐related target	FBXO10 overexpression suppresses hypertrophy and ferroptosis; ferroptosis inhibition rescues the aggravated phenotype after FBXO10 loss, and ACSL4 overexpression counteracts FBXO10 protection	[23]
AngII‐treated cardiomyocytes; TAC‐induced hypertrophy (mice)	Hypertrophic remodeling and ferroptosis‐associated injury occur together with altered mitochondrial regulation and reduced Etfa expression	Irx3 or Etfa overexpression attenuates hypertrophy and ferroptosis; Etfa knockdown diminishes the protective effect of Irx3, supporting an Irx3–Etfa regulatory axis	[53]

## 4 Astragaloside IV: Chemical Structure, Pharmacology, and Anti‐Hypertrophic Mechanisms

### 4.1 Chemical Structure and Pharmacokinetic Properties

Astragaloside IV (AS‐IV) is a cycloartane‐type triterpene saponin and a prominent active component of Astragalus species [25]. AS‐IV has been shown to have antioxidant, anti‐inflammatory, anti‐fibrotic, and metabolic‐regulating effects in experimental cardiovascular models [25, 58, 59]. Oral exposure to unchanged AS‐IV is limited. Caco‐2 studies indicate poor intestinal permeability [60], while rat pharmacokinetic studies reported low oral bioavailability [61]. Intestinal bacteria can convert AS‐IV into metabolites such as cycloastragenol [62, 63]. Recent work using human gut microbial preparations, bifidobacteria, and lactic acid bacteria further demonstrated species‐dependent pathways of AS‐IV biotransformation, although the contribution of these metabolites to cardiovascular efficacy remains unresolved [63]. The above results suggest that intestinal microbial transformation may affect the systemic distribution of orally administered AS‐IV; it is unknown whether the resulting metabolites contribute to the cardiovascular or anti‐ferroptotic effects of AS‐IV. Evidence obtained by purified AS‐IV should be distinguished from evidence obtained from complex Astragalus‐containing preparations. The pharmacokinetic deficiency will limit systemic exposure, and adjustments to dosing and formulation need to be made in the following studies. The pharmacokinetics and translation issues are shown in Figure 2.

**FIGURE 2:**
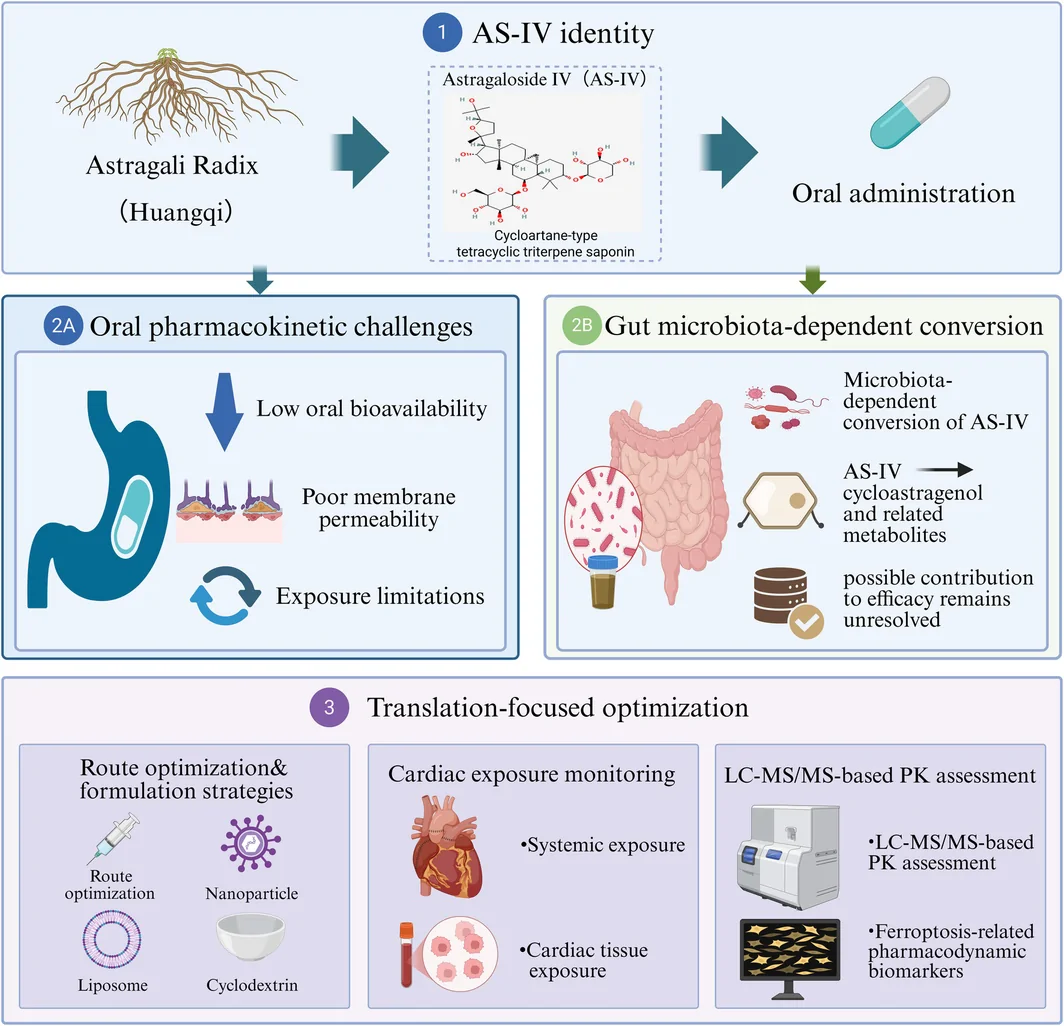
AS‐IV structure and translational pharmacokinetic challenges. The figure summarizes the cycloartane‐type saponin identity of AS‐IV and the principal translational challenges associated with oral administration, including limited oral bioavailability, poor intestinal and membrane permeability, and resulting exposure limitations. Gut microbiota‐dependent conversion of AS‐IV to cycloastragenol and related metabolites is shown as a pharmacokinetic consideration; the contribution of these metabolites to efficacy remains unresolved. Translation‐focused strategies include route and formulation optimization, LC–MS/MS‐based pharmacokinetic assessment, cardiac exposure monitoring, and measurement of ferroptosis‐related pharmacodynamic biomarkers. AS‐IV, astragaloside IV; LC–MS/MS, liquid chromatography–tandem mass spectrometry; PK, pharmacokinetics.

### 4.2 Established Anti‐Hypertrophic Actions: Evidence Across Multiple Experimental Models

Studies have documented the anti‐hypertrophic actions of AS‐IV across multiple experimental models. In mice subjected to surgical aortic constriction (a pressure‐overload model), AS‐IV suppressed cardiac enlargement, collagen deposition, and loss of function while decreasing cardiomyocyte death and inflammatory markers. These effects were associated with increased expression of the regulatory protein SIKE and reduced activation of the TBK1/PI3K/AKT cascade [26]. In cardiomyocytes with LPS‐induced hypertrophy, AS‐IV reduced cell size, actin‐fiber reorganization, and hypertrophic markers (ANP and BNP). AS‐IV reduced the LPS‐induced increase in resting intracellular Ca^2+^ and inhibited CaN/GATA‐4 activation and NFAT3 nuclear translocation. The reported effect of AS‐IV on resting intracellular Ca^2+^ was similar to that observed with verapamil [27]. A more recent LPS‐based study similarly reported reductions in cardiomyocyte hypertrophy and collagen‐related responses after AS‐IV treatment and implicated CCL2‐dependent NF‐κB signaling using transcriptomic analysis and pathway validation [64]. In rats and neonatal cardiomyocytes with isoproterenol‐induced hypertrophy, AS‐IV improved hemodynamic indices, reduced heart‐to‐body‐weight and left‐ventricle‐to‐body‐weight ratios, and suppressed ANP and BNP expression. AS‐IV also improved indices of myocardial energy metabolism, including the ATP/AMP ratio, and reduced circulating free fatty acids in association with modulation of NF‐κB/PGC‐1α signaling [28].

In hypoxia‐induced cardiac hypertrophy models in vivo and in H9C2 cells, AS‐IV attenuated hypertrophic changes in association with calpain‐1 suppression and restoration of mTOR phosphorylation [29]. AS‐IV also attenuated associated autophagic and apoptotic changes. In offspring born to dams exposed to intrauterine hypoxia, AS‐IV attenuated cardiac hypertrophy and improved hemodynamic abnormalities in association with inhibition of PKCβII/Egr‐1 signaling [30]. In experimental diabetic cardiac disease, AS‐IV improved systolic and diastolic function and reduced hypertrophy and fibrosis in association with suppression of calpain‐1/CaMKII signaling, Ca^2+^ overload, oxidative stress, and apoptosis. In diabetic mice, calpain‐1 knockout produced cardioprotective effects broadly similar to AS‐IV treatment, and additional AS‐IV treatment did not provide a clear further benefit in the knockout setting [31].

In a pressure‐overload mouse model, AS‐IV attenuated pathological myocardial hypertrophy and fibrosis in association with modulation of the EGR1‐SIRT1‐PPARα‐SCAD signaling pathway [32]. In a TAC‐induced pressure‐overload heart‐failure model, AS‐IV enhanced myocardial microvascular angiogenesis, reduced fibrosis, and improved cardiac function in association with HSF1/VEGF signaling; HSF1 downregulation attenuated these effects [65]. In a rat myocardial infarction model, AS‐IV was evaluated alone and in combination with tetramethylpyrazine. The study reported improvements in post‐infarction remodeling, including reduced fibrosis and remote‐myocardial hypertrophy and increased angiogenesis, with prominent effects in the combination‐treatment setting [66]. In a separate myocardial‐infarction model, AS‐IV reduced ventricular remodeling, myocardial fibrosis, and hypertrophic changes in association with reduced ROS/caspase‐1/GSDMD‐related pyroptotic signaling and inflammatory‐cell infiltration [67]. Pyroptosis is a distinct cell‐death mechanism relevant to broader cardiac remodeling and should not be conflated with ferroptosis. Integrated transcriptomic and proteomic analyses in an isoproterenol‐associated myocardial‐fibrosis model further linked the anti‐fibrotic effects of AS‐IV to reduced oxidative stress, cellular senescence, and p53‐associated signaling [68]. Recent heart‐failure studies have also associated AS‐IV‐mediated functional improvement with HIF/Rho/ROCK regulation [69] and with the SCAD/DJ‐1/SIRT1/FOXO3a axis linking energy metabolism, oxidative stress, and cardiomyocyte apoptosis [70]. These findings broaden the cardiac remodeling evidence base but do not establish a ferroptosis‐mediated anti‐hypertrophic mechanism.

Collectively, these experimental studies support multi‐pathway anti‐hypertrophic effects of AS‐IV involving calcium‐dependent signaling, stress‐response pathways, and metabolic regulation. Some of these pathways may intersect with ferroptosis‐related biology, but direct evidence that ferroptosis mediates these anti‐hypertrophic effects remains lacking. The experimentally reported anti‐hypertrophic actions of AS‐IV are summarized in Figure 3 and Table 2.

**FIGURE 3:**
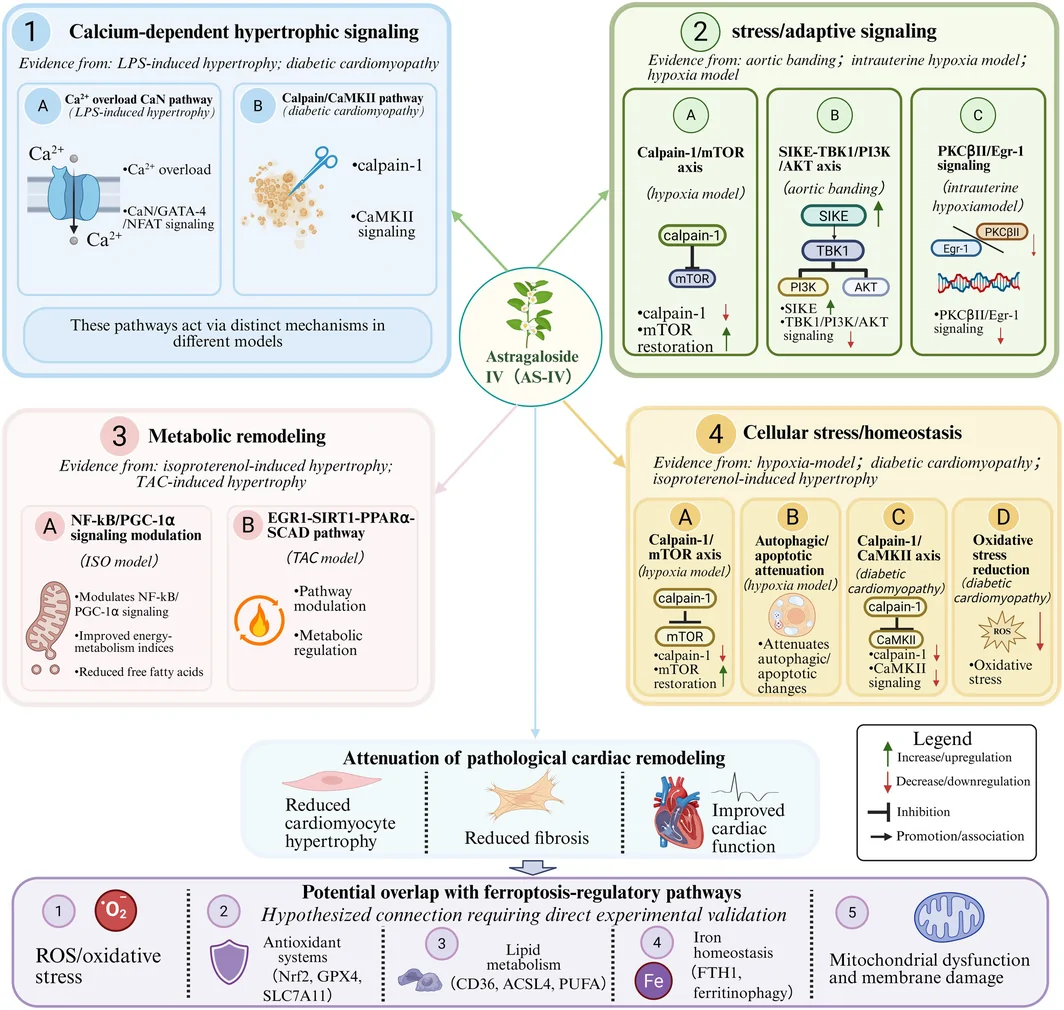
Multi‐target anti‐hypertrophic actions of Astragaloside IV (AS‐IV) in pathological cardiac remodeling. AS‐IV attenuates pathological cardiac hypertrophy through multiple experimentally reported mechanisms. Calcium‐dependent hypertrophic signaling includes suppression of Ca^2+^/CaN/GATA‐4/NFAT3 signaling in LPS‐induced hypertrophy and calpain‐1/CaMKII signaling in diabetic cardiomyopathy. Stress/adaptive signaling includes SIKE‐mediated modulation of TBK1/PI3K/AKT signaling in aortic banding, PKCβII/Egr‐1 signaling in the intrauterine‐hypoxia model, and calpain‐1/mTOR signaling in the hypoxia model. Metabolic effects include modulation of NF‐κB/PGC‐1α signaling in isoproterenol‐induced hypertrophy and the EGR1‐SIRT1‐PPARα‐SCAD pathway in TAC‐induced hypertrophy. Cellular stress/homeostasis effects include regulation of calpain‐1/mTOR and calpain‐1/CaMKII signaling, reduction of oxidative stress, and attenuation of autophagic and apoptotic changes in the hypoxia model. These established experimental actions converge on reduced cardiomyocyte hypertrophy and fibrosis and improved cardiac function. The potential overlap with ferroptosis‐regulatory pathways is shown as a hypothesized connection requiring direct experimental validation. ACSL4, acyl‐CoA synthetase long‐chain family member 4; AKT, protein kinase B; AS‐IV, astragaloside IV; CaMKII, calcium/calmodulin‐dependent protein kinase II; CaN, calcineurin; CD36, cluster of differentiation 36; Egr‐1/EGR1, early growth response 1; FTH1, ferritin heavy chain 1; GATA‐4, GATA binding protein 4; GPX4, glutathione peroxidase 4; ISO, isoproterenol; LPS, lipopolysaccharide; mTOR, mechanistic target of rapamycin; NFAT3, nuclear factor of activated T cells 3; NF‐κB, nuclear factor kappa B; Nrf2, nuclear factor erythroid 2‐related factor 2; PGC‐1α, peroxisome proliferator‐activated receptor gamma coactivator 1‐alpha; PI3K, phosphoinositide 3‐kinase; PKCβII, protein kinase C beta II; PPARα, peroxisome proliferator‐activated receptor alpha; PUFA, polyunsaturated fatty acid; ROS, reactive oxygen species; SCAD, short‐chain acyl‐CoA dehydrogenase; SIKE, suppressor of IKKε; SIRT1, sirtuin 1; SLC7A11, solute carrier family 7 member 11; TAC, transverse aortic constriction; TBK1, TANK‐binding kinase 1.

**TABLE 2:** Anti‐hypertrophic mechanisms of astragaloside IV across experimental models.

Model	Model type	Key mechanistic findings	References
Aortic banding (mice)	Pressure overload	SIKE elevation suppresses TBK1/PI3K/AKT signaling; hypertrophy, fibrosis, apoptosis, and inflammation attenuated	[26]
Lipopolysaccharide (neonatal rat cardiomyocytes)	Inflammatory/toxic	Resting Ca^2+^ reduced; CaN/GATA‐4 activation inhibited; NFAT3 nuclear translocation blocked	[27]
Isoproterenol (rats; NRVMs)	β‐adrenergic	NF‐κB/PGC‐1α‐regulated energy biosynthesis restored (ATP/AMP ↑, FFA ↓); ANP/BNP ↓	[28]
Hypoxia (cardiomyocytes)	Hypoxic	Calpain‐1 suppressed and p‐mTOR restored; hypertrophy, autophagy, and apoptosis attenuated	[29]
Intrauterine hypoxia (rats)	Developmental	PKCβII/Egr‐1 signaling inhibited; hemodynamics and cardiac mass indices improved	[30]
Diabetes (mice; H9c2, fibroblasts)	Metabolic	Calpain‐1/CaMKII suppressed; Ca^2+^ overload, apoptosis, and oxidative stress reduced; hypertrophy and fibrosis attenuated	[31]
Transverse aortic constriction (mice)	Pressure overload	EGR1‐SIRT1‐PPARα‐SCAD signaling modulated; hypertrophy and fibrosis attenuated	[32]
Coronary ligation‐induced myocardial infarction (rats; AS‐IV alone and with TMP)	Ischemic remodeling	Post‐infarction remodeling and Shh‐related signaling were improved, with prominent effects reported in the AS‐IV/TMP combination‐treatment setting	[66]
LPS‐treated H9C2 cardiomyocytes	Inflammatory	AS‐IV reduces hypertrophic and collagen‐related responses in association with CCL2‐dependent NF‐κB signaling regulation.	[64]
Myocardial infarction (mice)	Ischemic remodeling	Ventricular remodeling, myocardial fibrosis, and hypertrophic changes are attenuated in association with reduced ROS/caspase‐1/GSDMD‐related pyroptotic signaling	[67]

## 5 Astragaloside IV as A Ferroptosis Suppressor: Current Evidence Base

### 5.1 Direct Cardiac Evidence

Four published studies provide direct evidence that AS‐IV suppresses ferroptosis‐associated injury in cardiac disease models, although none directly tests pathological cardiac hypertrophy as the primary model. In adriamycin‐treated rats, AS‐IV improved cardiac injury and reduced fibrosis and oxidative stress while suppressing ferroptosis‐associated changes in association with Nrf2 activation and GPX4 upregulation [33]. This study provided early direct evidence linking AS‐IV to ferroptosis regulation in cardiac tissue.

In diabetic cardiomyopathy, AS‐IV improved myocardial injury and cardiac function while reducing myocardial lipid accumulation. In palmitate‐treated cardiomyocytes, AS‐IV decreased CD36 expression, lipid accumulation, lipid peroxidation, intracellular Fe^2+^ accumulation, and MDA, and reversed ferroptosis‐associated changes in ACSL4, p53, and GPX4. CD36 overexpression attenuated these protective effects, supporting a mechanistic role for CD36‐dependent lipid handling in AS‐IV‐mediated ferroptosis suppression [34].

In a chronic heart failure model after coronary ligation, bioinformatic target analysis and molecular modeling identified p53 as a potential hub linking AS‐IV to ferroptosis regulation. AS‐IV improved cardiac function and myocardial structure, reduced fibrosis and mitochondrial injury, decreased myocardial MDA and iron content, increased GSH, reduced p53 and phosphorylated p53, and increased SLC7A11 and GPX4 expression [35]. These findings support an association between p53/SLC7A11/GPX4 regulation and the anti‐ferroptotic effects of AS‐IV, although direct causal validation remains incomplete.

In cardiomyocytes subjected to anoxia/reoxygenation and in rats with myocardial ischemia–reperfusion injury, AS‐IV preserved cardiac function, reduced infarct‐related injury and oxidative stress, limited iron accumulation, and reversed ferroptosis‐associated changes in GPX4, PTGS2, MDA, and 4‐HNE. Mechanistically, AS‐IV increased 14‐3‐3η abundance and was associated with enhanced 14‐3‐3η–GPX4 interaction and regulation of GPX4 stability and ubiquitination, accompanied by preservation of mitochondrial integrity, including NDUFB8, UQCRC2, and membrane potential [36]. These findings support post‐translational regulation of GPX4 in this model without establishing an absolute degradation mechanism.

### 5.2 Evidence From Non‐Cardiac Organ Systems

Direct cardiac evidence supports AS‐IV‐mediated suppression of ferroptosis‐associated injury in several cardiac disease models, but direct evidence remains lacking in pathological cardiac hypertrophy models, and no study has yet shown that ferroptosis inhibition mediates the anti‐hypertrophic effect of AS‐IV. Non‐cardiac studies provide additional mechanistic support for conserved anti‐ferroptotic actions of AS‐IV, but should be interpreted as evidence of biological plausibility rather than proof of a cardiac hypertrophy‐specific mechanism.

After subarachnoid hemorrhage, AS‐IV reduced neuronal iron accumulation and lipid peroxidation, restored SLC7A11 and GPX4 expression, and activated Nrf2/HO‐1 signaling. Pharmacological inhibition of Nrf2 attenuated the protective effects of AS‐IV, supporting a functional role for this antioxidant pathway in ferroptosis suppression [37].

Similar findings have been reported in lung injury models. In mice exposed to PM2.5, AS‐IV reduced lung injury and ferroptosis‐associated changes through a mechanism involving the Nrf2/SLC7A11/GPX4 axis; erastin was used as a ferroptosis‐inducing intervention to test the involvement of this pathway [38]. AS‐IV has also been reported to attenuate ferroptosis‐associated oxidative and inflammatory injury in radiation‐induced lung injury [45]. Together, these studies support the recurrent involvement of antioxidant defense in the anti‐ferroptotic actions of AS‐IV, while the specific downstream pathways remain model dependent.

Kidney studies further suggest a potential link with intestinal microbiota. In diabetic nephropathy models, AS‐IV altered the intestinal microbiota and attenuated renal ferroptosis, supporting a potential gut microbiota–ferroptosis/gut–renal axis [41]. Orally administered AS‐IV has limited intestinal permeability and low oral bioavailability [60, 61], while intestinal bacterial conversion to cycloastragenol‐related metabolites has been reported separately [62]. Whether microbiota‐dependent metabolism directly contributes to the anti‐ferroptotic effects of AS‐IV remains unresolved.

Additional studies have reported AS‐IV‐mediated attenuation of ferroptosis‐associated injury in the liver, retina, prostate, cartilage, and other tissues [39, 40, 41, 42, 43, 44, 45, 46]. These findings provide cross‐organ mechanistic support but should not be treated as direct evidence for a cardiac‐specific or hypertrophy‐specific mechanism. Figure 4 summarizes the distinction between direct cardiac evidence and cross‐organ biological plausibility.

**FIGURE 4:**
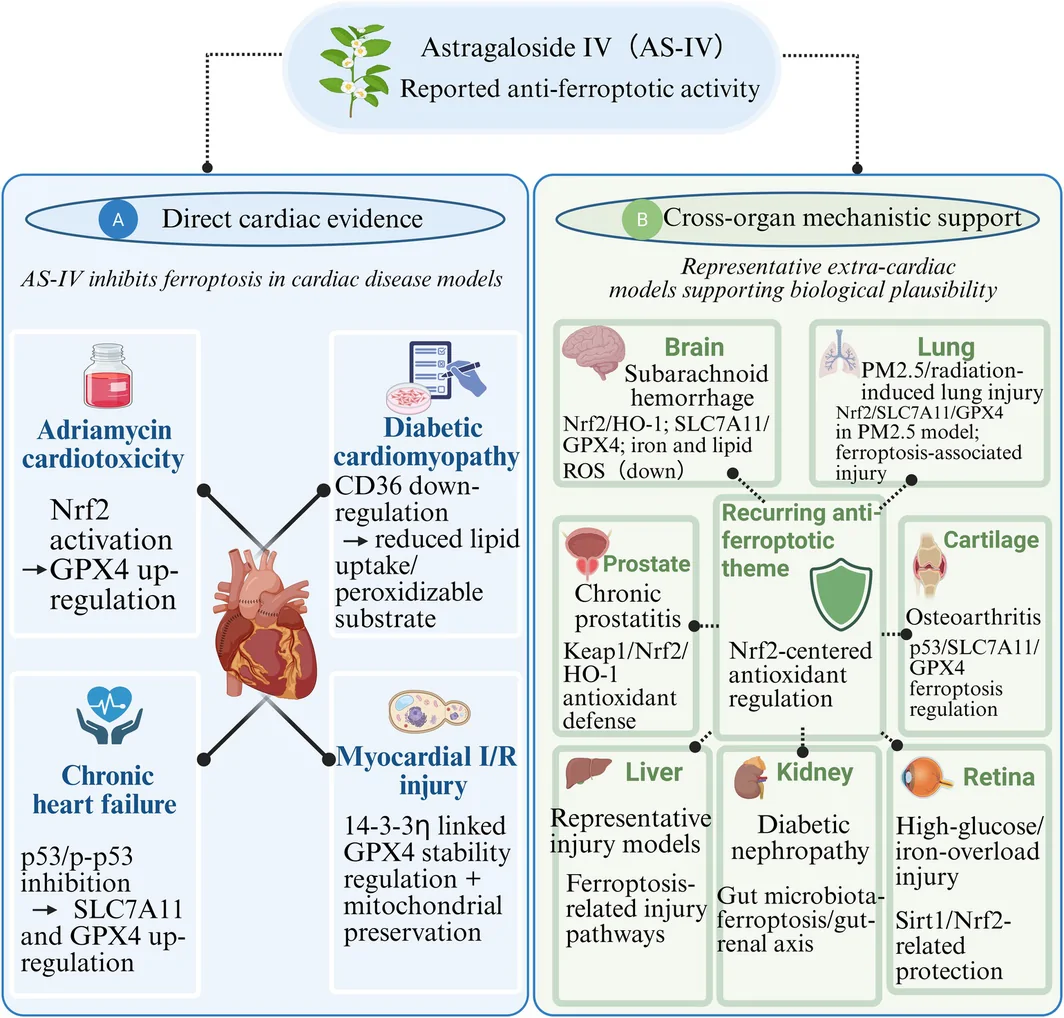
Evidence landscape of AS‐IV‐mediated ferroptosis regulation: Direct cardiac evidence versus cross‐organ mechanistic support. Direct cardiac evidence derives from adriamycin cardiotoxicity, diabetic cardiomyopathy, chronic heart failure, and myocardial ischemia–reperfusion injury. In myocardial ischemia–reperfusion injury, the evidence is summarized cautiously as 14‐3‐3η‐linked regulation of GPX4 stability together with mitochondrial preservation. Cross‐organ studies provide mechanistic support and biological plausibility rather than proof of a cardiac‐specific mechanism. Kidney evidence is presented as a potential gut microbiota–ferroptosis/gut–renal axis. In lung models, Nrf2/SLC7A11/GPX4 involvement is specified for PM2.5‐induced injury, whereas radiation‐induced lung injury is described more generally as ferroptosis‐associated injury; the two models are not assumed to validate an identical pathway. 14‐3‐3η, 14‐3‐3 eta protein; AS‐IV, astragaloside IV; CD36, cluster of differentiation 36; GPX4, glutathione peroxidase 4; HO‐1, heme oxygenase 1; I/R, ischemia/reperfusion; Keap1, Kelch‐like ECH‐associated protein 1; Nrf2, nuclear factor erythroid 2‐related factor 2; p53, tumor protein p53; PM2.5, particulate matter with an aerodynamic diameter of 2.5 μm or less; p‐p53, phosphorylated p53; ROS, reactive oxygen species; SIRT1, sirtuin 1; SLC7A11, solute carrier family 7 member 11.

## 6 Molecular Mapping: AS‐IV Targets Within the Ferroptosis Network

Based on the analysis of the available evidence, AS‐IV regulates ferroptosis susceptibility through several recurrent regulatory nodes (Figure 5 and Table 3).

1. Nrf2‐Orchestrated Antioxidant Defense. Nrf2 activation is a type of anti‐ferroptotic response to AS‐IV in several disease models. AS‐IV was associated with Nrf2 activation and GPX4 upregulation in adriamycin‐induced cardiac injury [33]. In subarachnoid hemorrhage and PM2.5‐induced lung injury, Nrf2‐associated protection involved SLC7A11/GPX4 and HO‐1 in the former model [37, 38]. Keap1/Nrf2/HO‐1 signaling was implicated in chronic prostatitis [42], whereas Sirt1/Nrf2‐related antioxidant regulation was reported in high‐glucose‐treated retinal pigment epithelial cells [43]. Functional Nrf2 involvement in iron‐overload retinal injury was supported by inhibitor experiments [44]. Together, these studies support recurrent Nrf2‐mediated antioxidant regulation, and the specific downstream effectors differ in different tissues and experimental conditions.
2. GPX4 Abundance and Functional Stability. AS‐IV may preserve GPX4 through mechanisms beyond Nrf2‐dependent transcription. AS‐IV was associated with 14‐3‐3η‐linked regulation of GPX4 stability and ubiquitination in myocardial ischemia–reperfusion injury [36]. In chondrocytes, p53 overexpression reversed key anti‐ferroptotic effects of AS‐IV, supporting p53‐dependent regulation of the SLC7A11/GPX4 axis [39]. In chronic heart failure, AS‐IV decreased p53 and phosphorylated p53, increased SLC7A11 and GPX4, but the causal relationship of this pathway has not been confirmed [35]. Together, these results indicate that GPX4 abundance and stability are repeatedly regulated nodes in AS‐IV‐mediated ferroptosis inhibition.
3. Lipid and Iron Substrate Availability. AS‐IV may also influence ferroptosis susceptibility by modifying lipid handling and iron availability. In diabetic cardiomyopathy, AS‐IV reduced CD36 expression, myocardial lipid accumulation, and ferroptosis‐associated injury, whereas forced CD36 overexpression attenuated these effects [34]. In several cardiac and extra‐cardiac ferroptosis models, AS‐IV has been associated with reduced iron accumulation and/or preservation of GSH, although the specific iron‐handling changes vary by tissue and model [35, 36, 37, 38, 42]. Changes in iron‐handling proteins appear to be model dependent and should not be generalized across tissues without direct validation.
4. Mitochondrial Function Preservation. Mitochondrial preservation frequently accompanies the anti‐ferroptotic effects of AS‐IV. During myocardial ischaemia‐reperfusion injury, AS‐IV maintained mitochondrial membrane potential, respiratory‐chain components NDUFB8 and UQCRC2, and mitochondrial structure [36]. Mitochondrial protection has also been observed in chronic heart failure associated with reduced ferroptosis‐related injury [35]. The above observations suggest that mitochondria may be involved in the anti‐ferroptotic effect of AS‐IV, but it has not been determined whether they are the cause.

**FIGURE 5:**
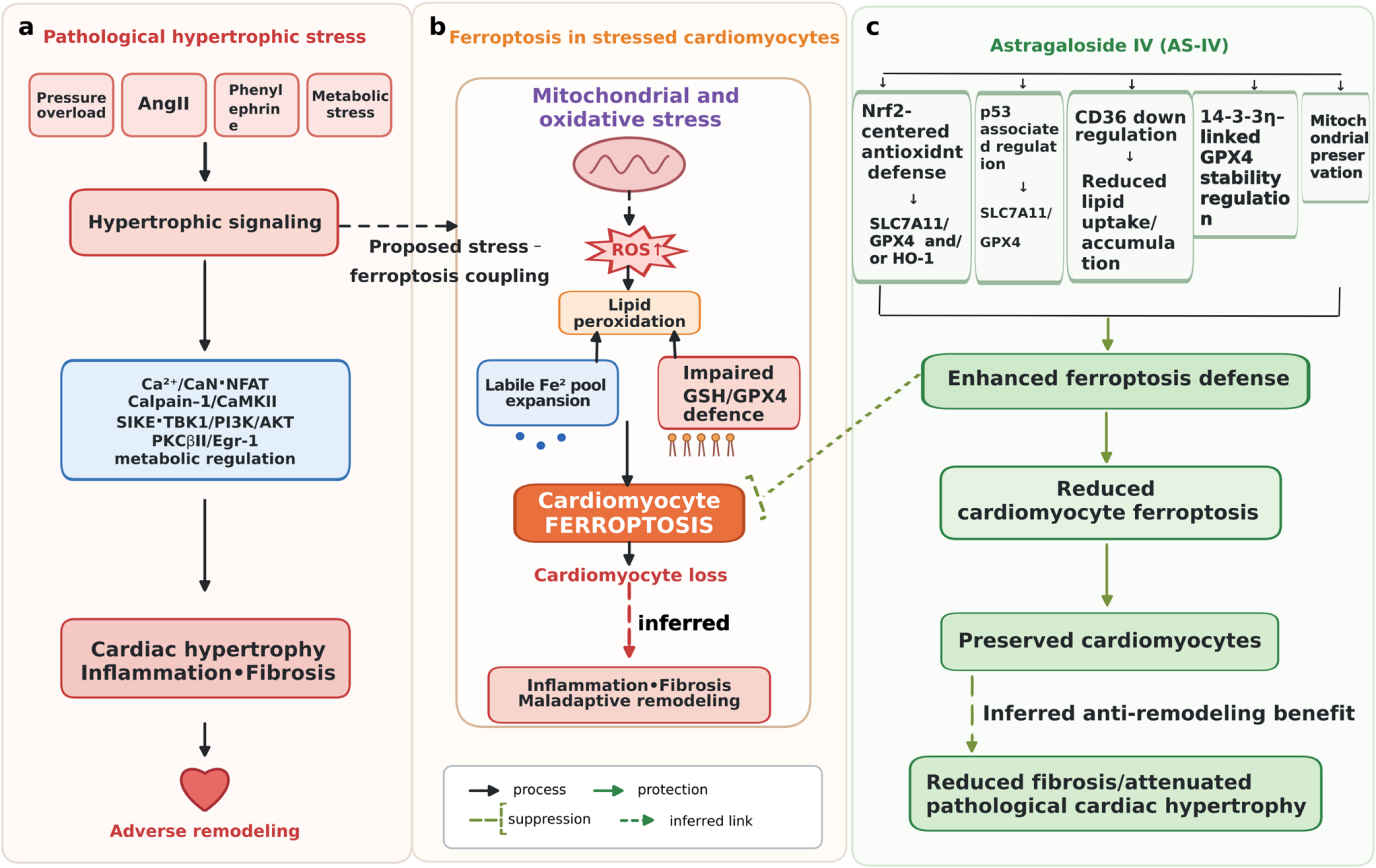
Integrated model linking AS‐IV, ferroptosis, and pathological cardiac hypertrophy. (a) Pressure overload, neurohormonal stimulation, and metabolic stress activate canonical hypertrophic signaling pathways and promote cardiac hypertrophy, inflammation, fibrosis, and adverse remodeling. The cross‐panel connection to ferroptosis represents a proposed stress–ferroptosis interface. (b) In stressed cardiomyocytes, mitochondrial and oxidative stress, expansion of the labile Fe^2+^ pool, impairment of GSH/GPX4‐dependent antioxidant defense, and lipid peroxidation can increase ferroptosis susceptibility. Cardiomyocyte ferroptosis causes cell loss and may contribute to inflammation, fibrosis, and maladaptive remodeling. (c) Evidence from cardiac and extra‐cardiac disease models indicates that AS‐IV modulates several anti‐ferroptotic nodes, including Nrf2‐centered antioxidant defense, p53‐associated SLC7A11/GPX4 regulation, CD36‐dependent lipid handling, 14‐3‐3η‐linked GPX4 stability regulation, and mitochondrial preservation. These actions converge conceptually on reduced ferroptosis and preservation of cardiomyocytes. AS‐IV‐mediated ferroptosis suppression leading to attenuation of pathological cardiac hypertrophy is the integrated hypothesis proposed in this review and remains to be directly validated in cardiac hypertrophy models. Solid arrows denote relationships supported within the cited disease models; dashed arrows denote inferred or hypothesized links, and dashed blunt‐ended lines denote inferred suppression. 14‐3‐3η, 14‐3‐3 eta protein; AKT, protein kinase B; AngII, angiotensin II; AS‐IV, astragaloside IV; Ca^2+^, calcium ion; CaMKII, calcium/calmodulin‐dependent protein kinase II; CaN, calcineurin; CD36, cluster of differentiation 36; Egr‐1, early growth response 1; Fe^2+^, ferrous iron; GPX4, glutathione peroxidase 4; GSH, glutathione; HO‐1, heme oxygenase 1; NFAT, nuclear factor of activated T cells; Nrf2, nuclear factor erythroid 2‐related factor 2; p53, tumor protein p53; PI3K, phosphoinositide 3‐kinase; PKCβII, protein kinase C beta II; ROS, reactive oxygen species; SIKE, suppressor of IKKε; SLC7A11, solute carrier family 7 member 11; TBK1, TANK‐binding kinase 1.

**TABLE 3:** Direct cardiac evidence that astragaloside IV inhibits ferroptosis.

Setting/model	Key ferroptosis and tissue readouts	Proposed mechanism	References
Adriamycin cardiotoxicity (rats, 35 days)	Cardiac ferroptosis attenuated; NOX2/NOX4 reduced; myocardial fibrosis and TGF‐β/collagen I/III‐associated fibrotic changes decreased	Nrf2 activation → GPX4 upregulation	[33]
Diabetic cardiomyopathy (rats; palmitate‐treated cardiomyocytes)	Myocardial lipid deposition and cardiomyocyte lipid accumulation reduced; ferroptosis‐associated alterations attenuated; contractile function improved	CD36 downregulation → reduced lipid uptake/accumulation and ferroptosis	[34]
Chronic heart failure after coronary ligation (rats; bioinformatics‐guided)	Myocardial MDA and Fe^2+^ decreased; GSH increased; mitochondrial damage reduced; p53/p‐p53 decreased; SLC7A11/GPX4 upregulated	p53/p‐p53 ↓ with SLC7A11/GPX4 ↑ (association; causal validation lacking)	[35]
Myocardial ischemia–reperfusion injury (H9c2 A/R; rat I/R)	ROS and Fe^2+^ decreased; GPX4 increased and PTGS2 decreased; MDA/4‐HNE reduced; mitochondrial function and integrity (NDUFB8, UQCRC2, ΔΨm) preserved	14‐3‐3η‐linked regulation of GPX4 stability and ubiquitination; mitochondrial preservation	[36]

## 7 Integrative Model: Convergence of AS‐IV Activity, Ferroptosis, and Cardiac Hypertrophy

### 7.1 Convergent Molecular Nodes

Based on the above evidence, two distinct but potentially related observations emerge: first, AS‐IV attenuates pathological cardiac hypertrophy in several experimental models; second, AS‐IV reduces ferroptosis‐related injury in multiple cardiac and extra‐cardiac disease models. These two effects may mechanistically overlap, but such a relationship has not been directly demonstrated in pathological cardiac hypertrophy models.

Pathological hypertrophic stimuli, such as mechanical overload, neurohormonal stimulation and metabolic dysfunction, activate the calcium‐calcineurin‐NFAT, calpain‐CaMKII, NF‐κB, mTOR and other stress‐response pathways [27, 28, 29, 30, 31]. Independently, in pathological hypertrophy models, ferroptosis‐related alterations also occur, such as lipid peroxidation, disrupted iron homeostasis, and reduced GSH/GPX4‐dependent antioxidant defense [11, 12, 15, 16, 17, 18]. AS‐IV modulates several recurrent ferroptosis‐related nodes in separate cardiac and extra‐cardiac disease models: (i) Nrf2‐centered antioxidant defense involving SLC7A11, GPX4, and/or HO‐1 [33, 37, 38, 42, 43, 44]; (ii) p53‐associated regulation of the SLC7A11/GPX4 axis [35, 39]; (iii) CD36‐dependent lipid uptake and accumulation [34]; and (iv) 14‐3‐3η‐linked regulation of GPX4 stability [36]. Together, the above observations provide a biological basis for the hypothesis that AS‐IV reduces ferroptosis susceptibility in hypertrophy. They have not shown that ferroptosis inhibition is responsible for the anti‐hypertrophic effect of AS‐IV.

Some mechanistic overlaps provide a reason to test the hypothesis. Calcium‐ and calpain‐dependent signaling is one example: AS‐IV modulates calcium‐calcineurin and calpain pathways in hypertrophy models [27, 29, 31], while independent cardiac studies link disrupted calcium handling and calpain activity to oxidative stress, mitochondrial dysfunction, and ferroptosis susceptibility. It has not been determined whether these pathways mechanistically link AS‐IV‐mediated hypertrophy attenuation to ferroptosis regulation.

Another possible intersection is metabolic remodeling. AS‐IV improves energy metabolism via the NF‐κB/PGC‐1α pathway in isoproterenol‐induced hypertrophy [28], and modulates the EGR1‐SIRT1‐PPARα‐SCAD signaling pathway in pressure‐overload hypertrophy [32]. These metabolic‐regulatory effects may change the lipid‐ and redox‐environment that promotes ferroptosis; however, this link has not been confirmed. Nrf2‐related antioxidant regulation is also associated with both hypertrophic stress and ferroptosis, but its specific role in AS‐IV‐mediated anti‐hypertrophic effects has not been determined.

In addition, it has been observed that some ferroptosis‐modulating treatments reduce both ferroptosis‐related damage and hypertrophy in experimental models. Paeonol, mocetinostat, and N‐Lac‐Phe attenuate hypertrophic phenotypes while reducing ferroptosis‐associated changes [15, 16, 18]. AS‐IV is also anti‐ferroptotic and cardioprotective in chronic heart failure, diabetic cardiomyopathy, anthracycline cardiotoxicity, and ischemia–reperfusion injury [33, 34, 35, 36]. The similarity of these phenotypes is only hypothesis‐generating and does not point to a common cause.

Another reason is that ferroptosis is a type of regulated cell death, and the continuous loss of cardiomyocytes can lead to adverse cardiac changes. Therefore, restricting ferroptosis injury may protect viable myocardium under chronic hypertrophic stress in theory. This possibility has not been directly verified in AS‐IV‐treated hypertrophy models.

### 7.2 Cell‐Type Specificity and the Fibrosis Dimension

Most of the direct hypertrophy‐related ferroptosis evidence reviewed here focuses on cardiomyocytes [11, 12, 15, 16, 17, 18]. Other populations of cardiac cells, such as vascular and immune cells, may also be involved in ferroptosis‐related cardiac injury; however, their roles in pathological hypertrophy remain unclear [12]. Fibroblasts are the primary effectors of myocardial fibrosis, but direct evidence linking fibroblast ferroptosis to the AS‐IV hypertrophy condition is still lacking. Recent human heart‐failure single‐cell analysis identified a fibroblast‐enriched ferroptosis‐associated transcriptional signature and a pseudotime trajectory linking ferroptotic stress with progressive fibroblast activation and pro‐fibrotic remodeling [71]. This human evidence strengthens the biological relevance of fibroblast ferroptosis in heart failure but does not demonstrate that fibroblast ferroptosis mediates AS‐IV‐induced anti‐remodeling effects. AS‐IV reduces myocardial fibrosis in several remodeling models [26, 31, 32, 66]. Recent systematic evaluation of cardiovascular fibrosis models supports reproducible anti‐fibrotic activity of AS‐IV across heterogeneous animal models, while also highlighting limitations in study quality, toxicological characterization, and clinical translation [72]. In adriamycin‐induced cardiac injury, fibrosis reduction occurs alongside suppression of ferroptosis‐associated changes and enhancement of Nrf2/GPX4 signaling [33], but a causal role for ferroptosis inhibition in the anti‐fibrotic effect has not been established. The extent to which non‐cardiomyocyte ferroptosis contributes to the overall anti‐remodeling effect of AS‐IV is still unknown.

## 8 Future Experimental Directions

The central hypothesis can be tested by the following experimental steps:

- AS‐IV and Ferroptosis in Hypertrophy Models. Evaluate AS‐IV in etiologically distinct hypertrophy models and assess hypertrophic remodeling in combination with a multi‐modal ferroptosis panel.
- Mechanistic Dependence. Employ cardiomyocyte‐specific, inducible, or partial loss‐of‐function strategies for GPX4/SLC7A11 defense or Nrf2 to determine whether ferroptosis regulation is necessary for the anti‐hypertrophic effect of AS‐IV.
- Ferroptosis specificity. Incorporate ferrostatin‐1 or liproxstatin‐1 and carefully titrated RSL3 or erastin in factorial experimental designs and simultaneously assess other cell death pathways.
- Causal Direction. In pre‐established hypertrophy, determine whether selective ferroptotic stress reduces the benefits of AS‐IV and whether ferroptosis‐selective rescue restores them while controlling for non‐specific toxicity.
- Reproducibility and Translation. Replicate the results in mechanistically different models to determine dose‐exposure relationships, systemic exposure, and cardiac exposure.

## 9 Discussion: Evidence Gaps, Technical Challenges, and Future Research Needs

### 9.1 Central Evidence Gap: Lack of Direct Hypertrophy Model Testing

To our knowledge, no study has directly investigated whether ferroptosis regulation mediates the anti‐hypertrophic effect of AS‐IV in an experimental model of pathological cardiac hypertrophy. Therefore, the evidence reviewed here falls into three categories: (1) ferroptosis‐related changes occur in pathological hypertrophy; (2) AS‐IV suppresses pathological hypertrophy via several known pathways; and (3) AS‐IV reduces ferroptosis‐related injury in other models of heart disease and in extra‐cardiac tissues. These three evidence branches remain experimentally distinct and do not establish that ferroptosis inhibition causally mediates the anti‐hypertrophic effects of AS‐IV. The experimental priorities required to test the above hypothesis are summarized in the preceding Section 8 and Figure 6.

**FIGURE 6:**
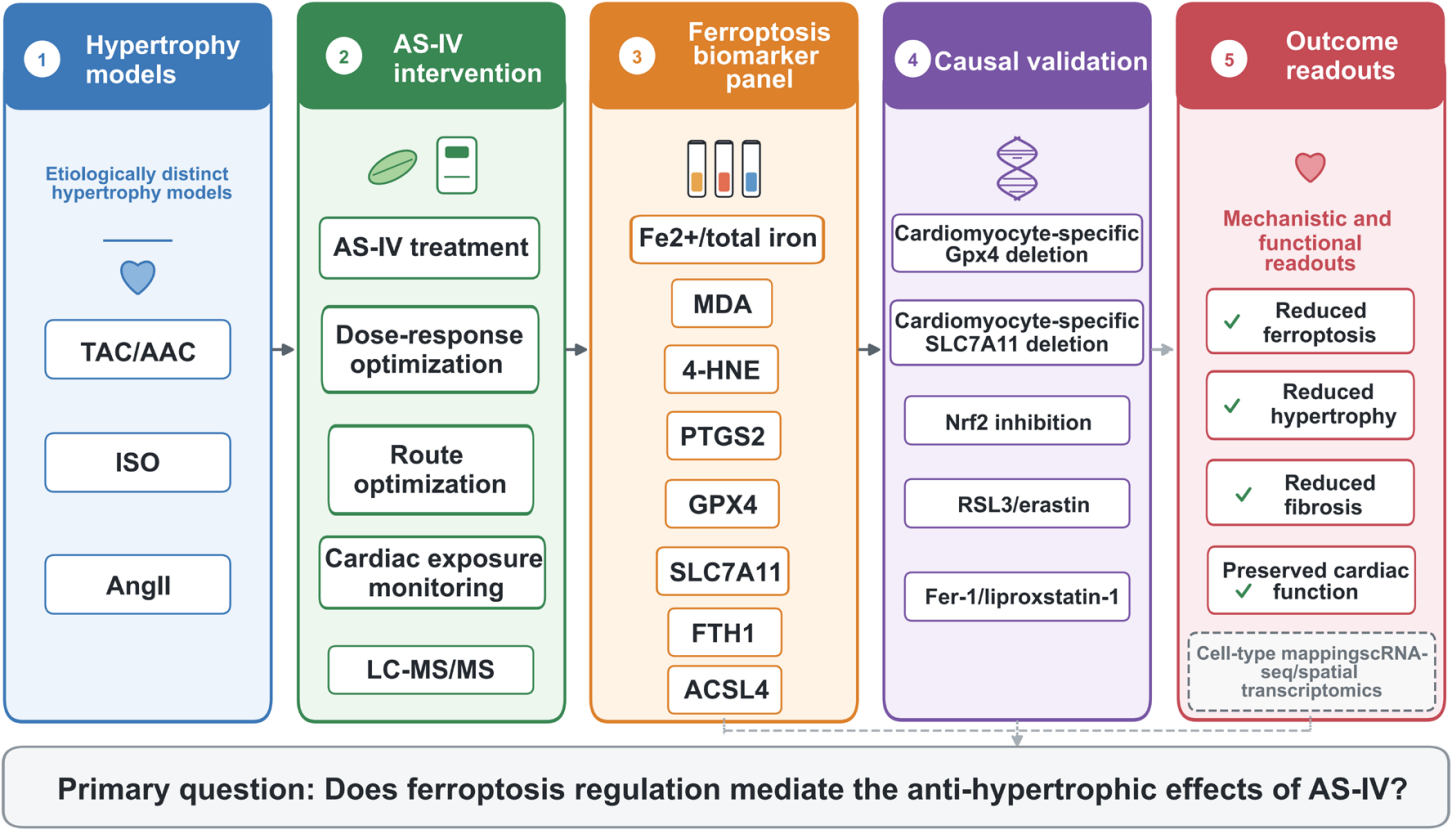
Experimental roadmap for validating the AS‐IV‐ferroptosis‐hypertrophy hypothesis. The proposed roadmap outlines a stepwise experimental strategy to directly test whether ferroptosis regulation mediates the anti‐hypertrophic effects of AS‐IV. Etiologically distinct hypertrophy models, including TAC/AAC, ISO, and AngII models, should be combined with AS‐IV dose–response and route optimization and LC–MS/MS‐based pharmacokinetic assessment, including cardiac exposure measurement where feasible. Ferroptosis assessment should use a multimodal panel integrating iron status (labile Fe^2+^ and/or total iron), lipid peroxidation (C11‐BODIPY, MDA, and 4‐HNE), antioxidant defense (GSH/GSSG, GPX4, and SLC7A11), and complementary ferroptosis‐associated proteins such as ACSL4, PTGS2, and FTH1. Causal validation should incorporate inducible or partial cardiomyocyte‐specific impairment of the GPX4/SLC7A11 defense system, Nrf2 loss‐of‐function approaches, carefully titrated ferroptosis induction with RSL3 or erastin, and ferroptosis inhibition with Fer‐1 or liproxstatin‐1. Outcome assessment should quantify ferroptosis, cardiomyocyte hypertrophy, fibrosis, cardiac function, and cell‐type‐specific responses, with single‐cell RNA sequencing and/or spatial transcriptomics used where feasible to map ferroptosis‐related transcriptional programs. The roadmap is intended to support or refute the proposed hypothesis and should not be interpreted as evidence that the mechanism has already been established. 4‐HNE, 4‐hydroxynonenal; AAC, abdominal aortic constriction; ACSL4, acyl‐CoA synthetase long‐chain family member 4; AngII, angiotensin II; AS‐IV, astragaloside IV; C11‐BODIPY, BODIPY 581/591 C11 lipid peroxidation‐sensitive fluorescent probe; Fe^2+^, ferrous iron; Fer‐1, ferrostatin‐1; FTH1, ferritin heavy chain 1; GPX4, glutathione peroxidase 4; GSH, glutathione; GSSG, glutathione disulfide; ISO, isoproterenol; LC–MS/MS, liquid chromatography–tandem mass spectrometry; MDA, malondialdehyde; Nrf2, nuclear factor erythroid 2‐related factor 2; PTGS2, prostaglandin‐endoperoxide synthase 2; RSL3, RAS‐selective lethal 3; scRNA‐seq, single‐cell RNA sequencing; SLC7A11, solute carrier family 7 member 11; TAC, transverse aortic constriction.

### 9.2 Technical Challenges in Ferroptosis Research

A Single Biomarker Cannot Be Used to Diagnose Ferroptosis. Lipid peroxidation products such as MDA and 4‐HNE are not exclusive to ferroptosis; therefore, interpreting them in conjunction with iron status, GPX4‐ and system xc‐related antioxidant defenses, morphology (where appropriate), and ferroptosis‐selective rescue or sensitisation experiments [4, 5, 6] strengthens the diagnosis of ferroptosis.

As the ferroptosis‐related biochemical changes may be time‐dependent, serial sampling should be employed when feasible.

The extent and timing of ferroptosis‐associated changes may differ among TAC, AAC, and neurohormonal hypertrophy models according to disease severity and sampling stage. Therefore, the experimental time and ferroptosis‐assessment panels need to be optimized for each model separately and cannot be used interchangeably. Iron loading should not be used solely to promote the detection of ferroptosis unless iron overload is the experimental condition.

Apoptosis, necroptosis, and ferroptosis may coexist during pathological remodeling. Therefore, the evidence of ferroptosis should be based on the convergence of biochemical and functional data, including targeted rescue when applicable, rather than a single marker.

### 9.3 Pharmacokinetics, Dosing, and Drug Delivery: Translating Bench Research to Clinical Application

Poor intestinal permeability and low oral bioavailability are serious translational limitations of AS‐IV [60, 61], and intestinal bacterial conversion to cycloastragenol‐related metabolites is another pharmacokinetic problem [62]. Recent reviews continue to address the problems of limited oral bioavailability and the need for improved pharmacokinetic characterization and drug‐delivery strategies in AS‐IV translation [58, 59]. As a proof‐of‐concept for formulation‐based delivery, a 2025 study developed a conductive bioadhesive hydrogel to achieve controlled myocardial delivery of AS‐IV after myocardial infarction. The platform increased local cardiac delivery and was associated with reduced ferroptosis‐related injury and improved post‐infarction repair [73]. As this intervention is an AS‐IV combined with a multi‐functional delivery scaffold, its results cannot be directly applied to free AS‐IV alone. In the future, the validated LC–MS/MS method will be employed to explore dose–exposure relationships in plasma and, where feasible, in cardiac tissue; studies will also determine whether microbial metabolites have pharmacological effects and investigate formulations or delivery systems that can increase systemic or cardiac exposure. Pharmacokinetic analysis and alterations in the redox‐iron system of the blood can be employed as pharmacodynamic indicators in clinical trials, but they are not direct indicators of myocardial ferroptosis.

### 9.4 Clinical Translation Within Traditional Medicine Contexts

AS‐IV is a known bioactive component of Astragalus species, but the evidence obtained from purified AS‐IV should be clearly distinguished from that obtained from Astragalus‐containing injections, herbal preparations or combination formulations. Clinical findings obtained from complex Astragalus‐containing preparations should not be taken as direct clinical evidence for purified AS‐IV. High‐quality prospective clinical trials of purified AS‐IV for pathological cardiac hypertrophy are unavailable. Recently, systematic reviews and meta‐analyses have also found that the cardiovascular evidence base for AS‐IV is still mainly preclinical. Meta‐analytic syntheses of experimental heart‐failure and ischemic‐heart‐disease models have shown improvements in cardiac function and remodeling, but these results cannot replace prospective clinical trials of purified AS‐IV [74, 75]. More and more foreign studies have also explored the applications of AS‐IV outside of East Asia; recently, groups in South and Southeast Asia/the Middle East, as well as European researchers, have focused on both its multi‐target pharmacology and the need for improved pharmacokinetic, safety and clinical data [75, 76]. Future translations will need standardized preparation, pre‐set dosages, pharmacokinetic characterization and clinically relevant structural and functional cardiac endpoints. Exploratory circulating measures of redox status and iron regulation may provide a mechanism but do not prove myocardial ferroptosis alone. Bioinformatic target‐network analysis and molecular modeling approaches similar to those in chronic heart failure can be applied to hypertrophy models for hypothesis generation and candidate selection [35]. Future multi‐omics studies will extend these computational methods.

### 9.5 Outstanding Conceptual Questions

Several conceptual questions remain unresolved. Most importantly, is ferroptosis suppression a mechanistic mediator of AS‐IV‐induced cardioprotection, or does it occur as a consequence of other antioxidant, anti‐inflammatory, or metabolic effects? Distinguishing causal mediation from a downstream association requires direct perturbation experiments. Other problems include whether prolonged exposure to AS‐IV changes iron regulation in the body, how ferroptosis of non‐cardiomyocytes contributes to damage, and how AS‐IV interacts with other cardiovascular drugs. The above questions will help determine whether the regulation of ferroptosis is a cause or a consequence of AS‐IV action.

This paper has some defects. It is a story and hypothesis‐generating synthesis rather than a systematic review or quantitative meta‐analysis, and relevant studies in unindexed sources or unpublished datasets may have been excluded. Recent systematic evaluations of AS‐IV in heart failure, ischemic heart disease and cardiovascular fibrosis have consistently found that it is mainly preclinical evidence; dosing and model design are heterogeneous, and standardized pharmacokinetic and translational validation are required [72, 73, 74, 75]. Most importantly, the central hypothesis that ferroptosis suppression mechanistically mediates the anti‐hypertrophic effects of AS‐IV remains untested. Cardiac evidence for AS‐IV‐mediated ferroptosis suppression comes from disease models other than dedicated pathological hypertrophy models, and extra‐cardiac studies provide biological plausibility rather than cardiac‐specific proof. Therefore, we will divide the proven results from the derived links and assumed predictions in the text. Publication bias in the natural‐product literature may also favor positive results, and negative or null studies of AS‐IV may be underrepresented.

## 10 Conclusion

Pathological cardiac hypertrophy is still a problem to be addressed, and although some research has shown that ferroptosis is involved, more studies are needed. AS‐IV has independently shown anti‐hypertrophic effects in multiple experimental models and anti‐ferroptotic activities in several cardiac disease models, such as anthracycline cardiotoxicity, diabetic cardiomyopathy, chronic heart failure, and ischemia–reperfusion injury. The two streams of evidence meet at biologically plausible regulatory nodes, such as Nrf2‐centric antioxidant defense, p53‐associated regulation of SLC7A11/GPX4, CD36‐mediated lipid handling, 14‐3‐3η‐linked regulation of GPX4 stability, and mitochondrial protection. However, current evidence does not establish that ferroptosis inhibition mediates the anti‐hypertrophic effects of AS‐IV. Therefore, we propose the above relationship as an empirical hypothesis rather than a known mechanism and present an experimental system to determine its causal effect directly in models of pathological cardiac hypertrophy. Validation or refutation of this hypothesis will help determine the mechanistic role of ferroptosis in AS‐IV‐mediated cardiac remodeling and guide future translational research.

## Author Contributions

**Pengbo Liu:** conceptualization, writing – original draft, methodology, visualization, data curation. **Yongzhi Deng:** writing – review and editing, resources, supervision, funding acquisition.

## Funding

This work was supported by the Traditional Chinese Medicine Research Fund of Shanxi Provincial Health Commission (No. 2025ZYYA016).

## Conflicts of Interest

The authors declare no conflicts of interest.

## Data Availability

The authors have nothing to report.
